# Variation in macroevolutionary dynamics among extant primates

**DOI:** 10.1002/ajpa.24622

**Published:** 2022-09-22

**Authors:** Jeremiah E. Scott

**Affiliations:** ^1^ Department of Medical Anatomical Sciences, College of Osteopathic Medicine of the Pacific Western University of Health Sciences Pomona California USA

**Keywords:** activity pattern, diversification, extinction, MiSSE, speciation

## Abstract

**Objectives:**

This study examines how speciation and extinction rates vary across primates, with a focus on the recent macroevolutionary dynamics that have shaped extant primate biodiversity.

**Materials and methods:**

Lineage‐specific macroevolutionary rates were estimated for each tip in a tree containing 307 species using a hidden‐state likelihood model. Differences in tip rates among major clades were evaluated using phylogenetic ANOVA. Differences among diurnal, nocturnal, and cathemeral lineages were also evaluated, based on previous work indicating that activity pattern influences primate diversification.

**Results:**

Rate variation in extant primates is low within clades and high between clades. As in previous studies, cercopithecoids stand out in having high net diversification rates, driven by high speciation rates and very low extinction rates. Platyrrhines combine high speciation and high extinction rates, giving them high rates of lineage turnover. Strepsirrhines and tarsiids have low rates of speciation, extinction, turnover, and net diversification. Hominoids are intermediate between platyrrhines and the strepsirrhine‐tarsiid group, and there is evidence for differentiation between hominids and hylobatids. Diurnal lineages have significantly higher speciation rates than nocturnal lineages.

**Conclusions:**

Recent anthropoid macroevolution has been characterized by marked variation in diversification dynamics among clades. Strepsirrhines and tarsiids are more uniform, despite divergent evolutionary and biogeographic histories. Higher speciation rates in diurnal lineages may be driven by greater ecological opportunity or reliance on visual signals for mate recognition. However, the differences among anthropoids indicate that factors other than activity pattern (e.g., clade competition, historical contingency) have had a more influential role in shaping recent primate diversification.

## 1 INTRODUCTION

A major goal of studies of primate biodiversity is to understand why species richness is unevenly distributed across the primate phylogenetic tree. One explanation that has intuitive appeal is that primate lineages vary in their rates of speciation and extinction. Such variation might be expected, given that primates have radiated across the tropics and subtropics of multiple biogeographic realms and have evolved an impressive array of ecological and morphological adaptations (Fleagle, 2013). However, in a classic paper on modeling the processes of lineage diversification, Raup et al. (1973) demonstrated using simulations that large differences in clade size and shape can easily arise among lineages with identical speciation and extinction probabilities simply by chance. This finding raises the possibility that some of the notable asymmetries in species richness observed in primate sister clades are a result of stochasticity in the same underlying macroevolutionary regime.

The first investigation to examine speciation and extinction rates in extant primates was conducted by Purvis et al. (1995). These authors detected rate heterogeneity by applying the birth–death model (e.g., Nee et al., 1994) to a time‐scaled supertree containing all taxa recognized at the species level at the time of their study (*n* = 203; Corbet & Hill, 1991). The main conclusion of Purvis et al. (1995) was that cercopithecoids differ from hominoids, platyrrhines, and strepsirrhines in having very high net rates of diversification (i.e., speciation rate minus extinction rate), indicating explosive growth over the last 10 million years of the clade's history. This result has been recovered in most subsequent studies of primate macroevolution and has survived substantial revisions to primate taxonomy and innovations in the methods used to reconstruct phylogeny and estimate macroevolutionary rates (Arbour & Santana, 2017; Fabre et al., 2009; Paradis, 1998). The macroevolutionary regime of cercopithecoids has also been detected in studies of trait‐dependent diversification, where it has been identified as an important confounding factor in attempts to link morphological and ecological traits to shifts in diversification among primates (FitzJohn, 2010; Paradis, 2005; Scott, 2018).

Whether other primates share a common macroevolutionary regime is an open question. There is some evidence that platyrrhines have higher net diversification rates than strepsirrhines (Purvis et al., 1995), and that certain platyrrhine clades may be similar to cercopithecoids (Fabre et al., 2009). This pattern of results is consistent with studies of trait‐dependent diversification that indicate that diurnal primates (mostly monkeys) have diversified at higher rates than nocturnal lineages (mostly strepsirrhines) (Magnuson‐Ford & Otto, 2012; Santini et al., 2015; Scott, 2018). Rate differences at smaller phylogenetic scales (e.g., within subfamilies) are also apparent in some analyses (Fabre et al., 2009; Paradis, 1998; Purvis et al., 1995), suggesting that variation in speciation and extinction probabilities has played an important role in structuring primate diversity at various levels of the phylogenetic hierarchy.

The present study uses species‐specific macroevolutionary rates to examine how diversification dynamics differ among extant primates. Species‐specific rates—also referred to as tip rates—characterize diversification dynamics at the terminal branches (i.e., tips) of a time‐scaled phylogeny (Maliet et al., 2019; Title & Rabosky, 2019; Vasconcelos et al., 2022a). Such rates provide estimates of present‐day processes while including some information on effects from the recent past that have shaped current regimes (within the last 10 million years; Rabosky et al., 2018; Schluter & Pennell, 2017). This emphasis on recent history mitigates some of the problems inherent in using molecular phylogenies to reconstruct diversification dynamics across the entire evolutionary history of a large, ancient clade, where temporal variation in rates and phylogenetically patterned extinction render model identifiability extremely challenging (Kubo & Iwasa, 1995; Louca & Pennell, 2020; Marshall, 2017; Morlon et al., 2022; Vasconcelos et al., 2022a). Thus, the focus of this study is on recent variation in macroevolutionary rates among the primate crown clades without attempting to reconstruct temporal changes in those rates or extend interpretations too far into the past. Understanding primate diversification dynamics in deep time requires direct evidence from the fossil record (Herrera, 2017a; Silvestro et al., 2019).

## 2 MATERIALS AND METHODS

### 2.1 Phylogenetic framework

Analyses were conducted using two molecular phylogenies from Springer et al. (2012), who generated four trees with the same topology, but which differ in how divergence dates were estimated. The two trees used here were time‐scaled under the assumption of autocorrelated rates of molecular evolution, with one tree having soft‐bounded constraints on fossil calibrations and the other having hard bounds. The other two trees from Springer et al. (2012) were time‐scaled under the assumption of independent rates of molecular evolution and are not considered here because independent rates are less biologically realistic than autocorrelated rates and they do not fit the primate data as well (dos Reis et al., 2018). In practice, studies that have used all four trees to examine macroevolutionary patterns have obtained similar signals across the trees (Arbour & Santana, 2017; Herrera, 2017a). The two trees used in the present analysis also produced very similar patterns, and therefore, only results generated using the tree with soft bounds are described in detail in the main text. Results for the tree with hard bounds are presented in the Supporting Information, except to note where the two sets of results diverge.

Each of the trees from Springer et al. (2012) contains 367 primate species. Prior to analysis, all taxa not recognized at the species level by Groves (2005) were pruned along with the five outgroup species, reducing the trees to 307 tips. The modified trees are 82% complete according to Groves's taxonomy (Table 1). The number of primate taxa recognized by primatologists as full species has been steadily rising since the early 1980s (Rylands & Mittermeier, 2014), and recent estimates of extant primate biodiversity exceed 500 species. The list published by Estrada et al. (2017) contains 504 species (not including humans). If that number is correct, then the phylogenetic framework used here is 61% complete. Analyses were initially conducted using Groves's estimate of primate species diversity to compute the sampling fraction used to estimate macroevolutionary rates (FitzJohn et al., 2009). Analyses were then repeated using the sampling fraction based on Estrada et al.'s count. Both sampling fractions produced similar patterns. Results from the analyses conducted using the Groves sampling fraction are reported in the main text; the results generated using the Estrada et al. sampling fraction are presented in the Supporting Information, with differences between the two sets of results noted in the main text where relevant.

**TABLE 1:** Differences in the number of primate species recognized by Groves (2005) and Estrada et al. (2017) for selected primate clades

Clade	Groves (2005)		Estrada et al. (2017)	
	*n*	*f*	*n*	*f*
Lorisiformes	28	0.821	34	0.676
Lemuriformes	60	0.867	103	0.505
Tarsiidae	7	0.714	11	0.455
Platyrrhini	128	0.719	171	0.538
Hominoidea	21	1.000	26	0.801
Cercopithecoidea	132	0.864	160	0.713
Primates	376	0.816	505	0.608

Abbreviations: *f*, sampling fraction, the number of species included in this study divided by the number of species recognized in each taxonomy; *n*, number of species recognized in a given taxonomy.

### 2.2 Parameter estimation using the MiSSE model

Tip rates for all species in the primate tree were estimated using the MiSSE model (missing state speciation and extinction; Vasconcelos et al., 2022a). MiSSE is part of a family of models used to investigate correlations between various types of traits and macroevolutionary rates (e.g., Beaulieu & O'Meara, 2016; Caetano et al., 2018; FitzJohn, 2010, 2012; Maddison et al., 2007; Magnuson‐Ford & Otto, 2012). The closely related HiSSE model (hidden state speciation and extinction) uses a hidden Markov process to model rate variation caused by unobserved factors (hidden states) that can confound tests for an association between a focal (observed) trait and diversification (Beaulieu & O'Meara, 2016). MiSSE is an extension of HiSSE in that it dispenses with observed traits and uses only hidden states to estimate macroevolutionary rates at the tips of a time‐scaled phylogeny (Vasconcelos et al., 2022a). Thus, MiSSE is a trait‐free alternative to related models that is appropriate when the objective is to estimate rates without regard to a specific trait. Tip rates estimated by MiSSE have the advantage of being model‐based, which makes them less subject to stochasticity and thus more accurate than nonparametric tip‐rate statistics that only quantify speciation rates (Maliet et al., 2019; Rabosky, 2018, 2019; Title & Rabosky, 2019; Vasconcelos et al., 2022a). In comparison to BAMM (Bayesian analysis of macroevolutionary mixtures; Rabosky, 2014), a widely used model‐based approach, MiSSE may be more sensitive to rate variation among lineages and has an underlying model of rate heritability, which allows rates to vary continuously rather than changing abruptly (i.e., shifting) as in BAMM, though such changes are still possible (Vasconcelos et al., 2022a).

The MiSSE analysis was conducted in R version 4.1.2 (R Core Team, 2021) using the package *hisse* (Beaulieu & O'Meara, 2016). The current implementation of MiSSE allows up to 26 hidden states, each with an associated turnover rate (*τ*) and extinction fraction (*ε*). These two parameters are functions of speciation rate (*λ*) and extinction rate (*μ*): *τ* = *λ* + *μ* and *ε* = *μ*/*λ*. Thus, turnover rate is the number of macroevolutionary events per lineage per unit time, and extinction fraction is the extinction rate relative to the speciation rate. Note that *λ* and *μ* can both be obtained from τ and ε (for equations, see Beaulieu & O'Meara, 2016). The fifth macroevolutionary parameter examined here is net diversification rate: *r* = *λ* − *μ*. This parameter describes how quickly a clade is expanding (*r* > 0) or contracting (*r* < 0).

Given the size of the primate tree, an exhaustive search among all possible MiSSE models (26 hidden states, 52 estimated diversification parameters) is not justified; the vast majority of models are far too complex for a tree containing only 307 species. Based on previous experience with the trees used here (Scott, 2018, 2019), I restricted the search to models with no more than four hidden states and seven estimated parameters. As an example, one of the most complex models had four turnover rates and three extinction fractions, with two of the hidden states constrained to have the same extinction fraction. I used the search algorithm available in *hisse* (function *MiSSEGreedy*) to evaluate models in chunks of seven. The algorithm assesses the models in each chunk using Akaike's information criterion (AICc), beginning with simpler models and then moving to similar models with greater complexity. The algorithm ends the search if all the models in a chunk are 10 or more AICc units away from the current best‐fitting model (i.e., ΔAICc ≥10). After the search, model sets were pruned (function *PruneRedundantModels*) of complex models with log‐likelihoods that were virtually identical to those of simpler models to reduce model redundancy.

Each model was used to reconstruct rates across the trees (function *MarginReconMiSSE*). Each node or tip has a marginal probability of being in each of the hidden states detected by a model; the rates reconstructed for a particular node or tip reflects those probabilities. The final reconstructed tip rates reported here are the rates obtained from averaging across all of the models in the model set (function *GetModelAveRates*), with each model's rate estimate for a given tip being weighted by its Akaike weight. The best‐fitting model (ΔAICc = 0) has the highest weight; the worst‐fitting models (ΔAICc >10) contribute very little to the model‐averaged rates. Tip rates generated from both trees and sampling fractions are presented in Table S1.

### 2.3 Inferences with phylogenetic ANOVA

Given the historical importance of major primate clades as analytical units in studies of primate macroevolution (Purvis et al., 1995), I tested for differences in tip rates among cercopithecoids, hominoids, platyrrhines, tarsiids, lorisiforms, and lemuriforms (including *Daubentonia*) using simulation‐based phylogenetic ANOVAs (Garland et al., 1993). These tests were conducted in R using *phytools* (Revell, 2012). *P*‐values were obtained from 10,000 simulations, and the sequential Bonferroni procedure was used to adjust for multiple comparisons (Rice, 1989).

In addition to documenting phylogenetic patterns, previous studies have also used primates as a model system for investigating trait‐dependent diversification (FitzJohn, 2010, 2012; Gómez & Verdú, 2012; Magnuson‐Ford & Otto, 2012; Paradis, 2005; Rolland et al., 2014; Santini et al., 2015). Although several traits have been implicated as sources of rate heterogeneity among primates, most of them—body size, diet, sociality, mating system, and geographic distribution—are uncorrelated with diversification in analyses that account for the high net diversification rates of cercopithecoids (Arbour & Santana, 2017; FitzJohn, 2010; Scott, 2018). Only activity pattern has withstood further scrutiny (Scott, 2018, 2019). As part of this analysis, I tested for differences in tip rates among activity patterns, with the main prediction being that diurnal species will have higher net diversification rates, speciation rates, and turnover rates than nocturnal species (Anderson & Wiens, 2017; Scott, 2018). Previous studies treated activity pattern as either a binary trait (diurnal vs. nocturnal) because of methodological constraints, thus lumping cathemeral species with one of the other groups (Magnuson‐Ford & Otto, 2012; Scott, 2018, 2019), or as a multistate character with a separate state for cathemeral species (Santini et al., 2015). The latter approach yielded evidence that cathemeral species have macroevolutionary dynamics that are distinct from those of diurnal and nocturnal lineages (Santini et al., 2015), but this conclusion has been questioned (Scott, 2018). I evaluated this hypothesis here by testing for differences in tip rates among diurnal, nocturnal, and cathemeral lineages. Classifications were taken primarily from Santini et al. (2015). A few species that are not present in Santini et al.'s published data set could be easily categorized based on the activity patterns of their close relatives.

### 2.4 A caveat about extinction estimates

In principle, extinction rates can be recovered from molecular phylogenies. Extinct species cannot be observed on such trees in most cases, but extinction is expected to leave characteristic signatures on tree shape and the temporal distribution of branching events, allowing inferences about extinction rates (Nee, Holmes, May, & Harvey, 1994a). In practice, however, extinction rates and extinction fractions have proven difficult to estimate with accuracy and precision, especially in the presence of rate heterogeneity through time and across lineages (Rabosky, 2010, 2016). Despite this difficulty, net diversification rates and turnover rates, which are functions of speciation and extinction rates, can be estimated with greater confidence (e.g., Beaulieu & O'Meara, 2016; Title & Rabosky, 2019). The extinction rates and extinction fractions presented here should be interpreted cautiously, and differences among groups in these two parameters should be considered hypotheses to be evaluated against the fossil record.

## 3 RESULTS

### 3.1 Overview

**FIGURE 1:**
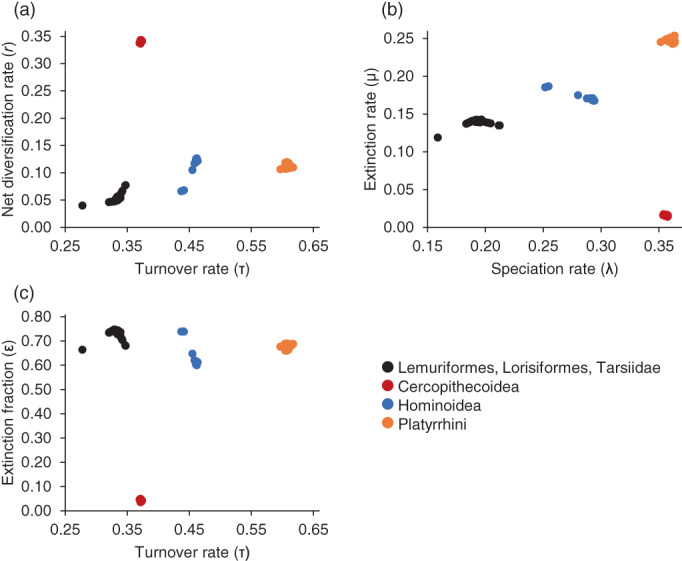
Tip rates estimated by the MiSSE analysis using the tree with soft‐bounded constraints and the sampling fraction derived from the taxonomy of Groves (2005)

As expected, the most prominent feature of recent primate macroevolution recovered by MiSSE is the very high net diversification rates of cercopithecoids relative to other extant primates (Figure 1a). There is also evidence for differences in macroevolutionary dynamics among other groups. A few general patterns are evident. First, lemuriforms, lorisiforms, and tarsiids are very similar to each other for all parameters. Second, speciation and extinction rates increase from strepsirrhines and tarsiids to hominoids to platyrrhines, with a concomitant increase in turnover rates, but cercopithecoids do not follow this trend (Figure 1b). Third, extinction rates in strepsirrhines, tarsiids, hominoids, and platyrrhines are high relative to speciation rate (extinction fraction > 0.60), whereas cercopithecoids have very low relative extinction rates (Figure 1c).

**FIGURE 2:**
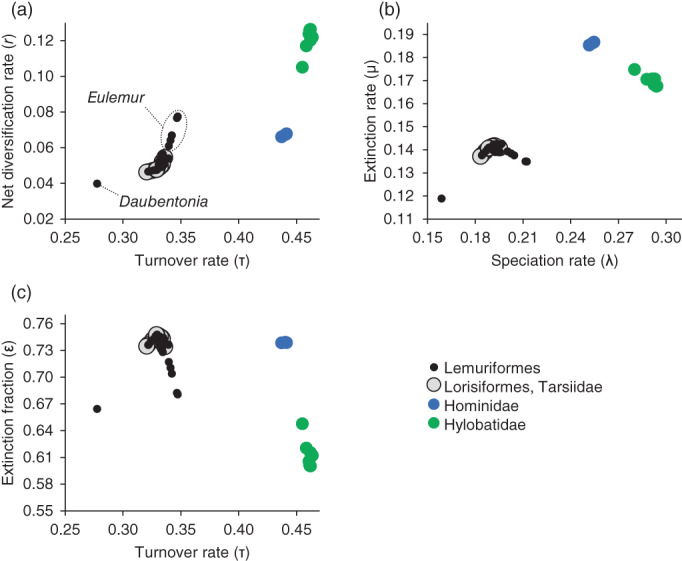
Same plots as in Figure 1, but zoomed in to highlight the variation within hominoids and lemuriforms, and the similarity of lemuriforms, lorisiforms, and tarsiids; species of *Eulemur* and *Daubentonia* are indicated in 2a, but are also differentiated from the main lemuriform clusters in 2b and 2c

Estimated tip rates exhibit strong phylogenetic signal. Cercopithecoids, platyrrhines, lorisiforms, and tarsiids have very little within‐clade variation. In contrast, hominoids and lemuriforms exhibit some internal structure. Most lemuriforms form a tight cluster, but species of *Eulemur* exhibit varying degrees of differentiation from other species, and *Daubentonia madagascariensis* is distinct (Figure 2). In hominoids, the elevated variation is driven by clear separation between hylobatids and hominids for some parameters. Given that the two hominoid clades form discrete clusters, I treated them as separate units in the phylogenetic ANOVAs reported below.

### 3.2 Differences among clades

Most of the differences evident in Figures 1 and 2 are statistically significant according to the phylogenetic ANOVAs (Table S2). The high net diversification rates estimated for cercopithecoids are the product of high speciation rates and low extinction rates in comparison to other primates, resulting in very low extinction fractions. Platyrrhines also have high speciation rates: depending on the sampling fraction used, platyrrhine speciation rates are as high as those of cercopithecoids (Groves sampling fraction; Figure 1a) or significantly higher (Estrada et al. sampling fraction). Either way, platyrrhines combine high speciation rates with high extinction rates, giving them the highest rates of lineage turnover among primates. Platyrrhines also have net rates of diversification that are higher than those of hominids, strepsirrhines, and tarsiids.

Lemuriforms, lorisiforms, and tarsiids cannot be statistically distinguished for any of the macroevolutionary parameters. These three clades have low rates of speciation, extinction, net diversification, and turnover compared to most anthropoids. The exceptions are (i) cercopithecoids, which, as noted, have lower rates of extinction, and (ii) hominids, which have similar net diversification rates. Note that this correspondence occurs despite significant differences in speciation and extinction rates, which are higher in hominids. Likewise, strepsirrhines, tarsiids, hominids, and platyrrhines all have similar extinction fractions despite having different rates of speciation and extinction.

The phylogenetic ANOVAs support the presence of distinct diversification dynamics within hominoids. In comparison to hominids, hylobatids have significantly higher rates of speciation, net diversification, and turnover, and lower extinction rates and extinction fractions. Whereas hominids resemble strepsirrhines and tarsiids in net diversification rate, hylobatids are similar to platyrrhines (Figure 1a).

### 3.3 Differences among activity patterns

**FIGURE 3:**
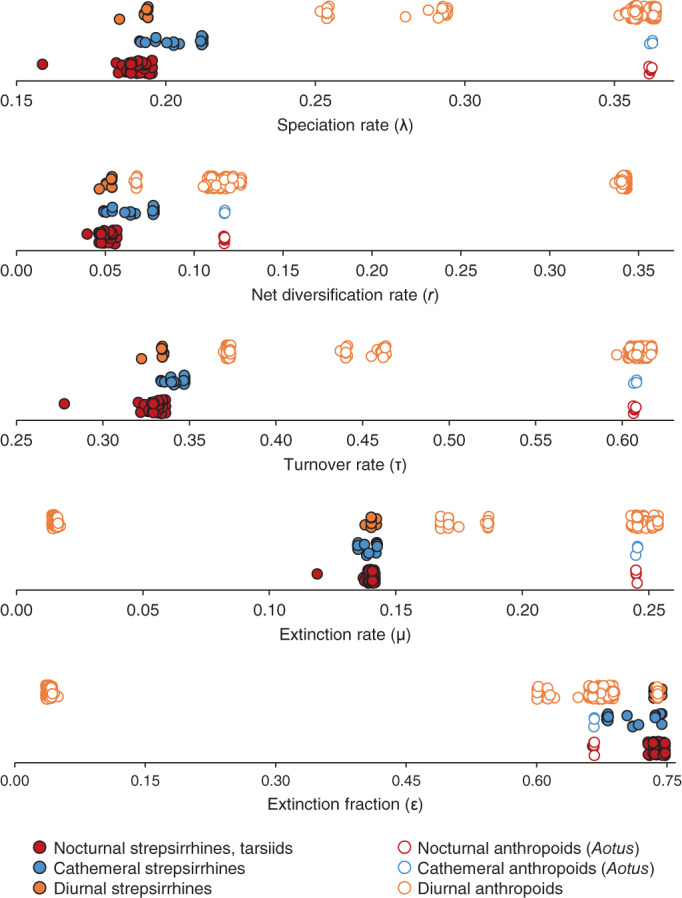
Tip rates plotted by activity pattern

The strong phylogenetic signal in macroevolutionary rates noted above is evident when species are classified by activity pattern (Figure 3). Nocturnal and cathemeral species of *Aotus* have rate estimates that are typical of diurnal platyrrhines. Diurnal and especially cathemeral strepsirrhines are shifted in the direction of diurnal anthropoids for some parameters, but they are most similar to nocturnal strepsirrhines and tarsiids.

Phylogenetic ANOVAs indicate that diurnal species have significantly higher speciation rates than nocturnal species, regardless of how cathemeral species are treated (Table S3). Diurnal and nocturnal species cannot be distinguished for any of the other macroevolutionary parameters, however. When cathemeral species are treated as a separate group, they are not significantly different from diurnal or nocturnal species for any comparison, with one exception: in the analysis that used the tree with hard‐bounded constraints and the sampling fraction based on the taxonomy of Groves (2005), cathemeral species, like nocturnal species, have significantly lower rates of speciation than diurnal species.

## 4 DISCUSSION

### 4.1 Recent diversity in primate diversification

The results of this study support the idea that current patterns of primate biodiversity have been shaped by heterogeneity in rates of speciation and extinction among clades. Previous studies have emphasized the high net diversification rates of cercopithecoids relative to other primates (Paradis, 1998; Purvis et al., 1995), driven by high speciation rates and very low extinction rates (Arbour & Santana, 2017). This signal was also detected here, along with four other macroevolutionary regimes. Lemuriforms, lorisiforms, and tarsiids differ from anthropoids and resemble each other by combining low rates of speciation, extinction, net diversification, and turnover. Platyrrhines have high rates of speciation, extinction, and turnover, and net rates of diversification that are elevated relative to strepsirrhines and tarsiids. Hominids and hylobatids are intermediate between platyrrhines and the strepsirrhine‐tarsiid group for most parameters, and they differ from each other: hylobatids have higher speciation rates, lower extinction rates, and, therefore, higher net diversification rates in comparison to hominids.

The pronounced differences among anthropoid clades are a striking contrast to the limited variation found within and between lemuriforms, lorisiforms, and tarsiids. Herrera (2017b) found mixed support for higher rates of speciation in lemuriforms relative to lorisiforms using a different phylogenetic framework and different methods. The results of the present study and Herrera's agree where the approaches are conceptually similar—i.e., where the models were not supplied with a priori information indicating where shifts in macroevolutionary dynamics were expected to occur. Given their disparate evolutionary and biogeographic histories, we might expect some differentiation among lemuriforms, lorisiforms, and tarsiids. Tarsiids last shared an ancestor with the two strepsirrhine clades in the Paleocene or earlier, and the split between lemuriforms and lorisiforms probably occurred by the early Eocene (dos Reis et al., 2018; Springer et al., 2012). Together, these three clades exhibit substantial adaptive diversity in their locomotor, digestive, and sensory systems (Fleagle, 2013; Fleagle & Reed, 1996). They also have distinctive geographic distributions and thus vary in their climatic, faunal, and floristic contexts (Kamilar, 2009; Reed & Bidner, 2004). Nevertheless, lemuriforms provide the only hints of rate heterogeneity in this group: relative to other lemuriforms, *Daubentonia* has lower rate estimates for all macroevolutionary parameters, while species of *Eulemur* tend to have higher speciation and net diversification rates and lower extinction fractions (Figure 2). These differences are not large, however, and should be interpreted with caution, especially in the case of *Daubentonia*—a single lineage that diverged from other lemuriforms approximately 50 million years ago (dos Reis et al., 2018; Springer et al., 2012).

A major feature uniting lemuriforms, lorisiforms, and tarsiids, and distinguishing them from anthropoids, is nocturnality. Only a handful of lemuriform species are diurnal or cathemeral, whereas nocturnality and cathemerality are restricted to a single anthropoid genus (Fleagle, 2013; Santini et al., 2015). Previous studies of trait‐dependent diversification have shown that activity pattern is correlated with macroevolutionary dynamics in primates (Magnuson‐Ford & Otto, 2012; Santini et al., 2015; Scott, 2018, 2019), whereas other traits that tend to distinguish strepsirrhines and tarsiids from anthropoids, such as body size, sociality, and geographic distribution, do not (Arbour & Santana, 2017; FitzJohn, 2010; Scott, 2018). The relatively homogeneous rates observed across strepsirrhines and tarsiids, and the tendency for anthropoids to have higher rates of speciation, extinction, net diversification, or turnover, are consistent with an activity‐pattern effect, as are the elevated rates of speciation and net diversification estimated for species of *Eulemur*—which are cathemeral—relative to the lemuriform background rates. On the other hand, activity pattern has no explanatory power within Anthropoidea, where rate variation is high but all taxa, with the exception of species of *Aotus*, are diurnal.

Because the anthropoid clades show high between‐group variation and low within‐group variation, the causal mechanisms driving these rate differences will be difficult to identify. Possible candidates include clade‐specific traits, biogeographic effects, interactions between clades, and regional differences in how global climatic trends have shaped primate habitats. The strong phylogenetic signal in macroevolutionary rates also suggests a major role for historical contingency. For example, the decline of hominoid diversity that began in the late Miocene and the coincident expansion of cercopithecoids (Andrews, 1981; Fleagle, 2013; Hunt, 2016) are likely to have had an impact on variation in tip rates among extant catarrhines. Turnover in catarrhine diversity has been linked to climatic cooling and its sequelae, with the contrasting dietary, locomotor, and life‐history adaptations of hominoids and cercopithecoids mediating their divergent macroevolutionary responses to colder, drier conditions, increasing seasonality, and the spread of woodlands and grasslands following the Miocene Climatic Optimum (Hlusko et al., 2016; Kelley, 1997; Lambert, 2015; Scott, 2018). This scenario implicates the timing of events and the interaction between hominoids and cercopithecoids as important factors for explaining rate variation among catarrhines: some of the differences in rates may be related to the recency of the crown cercopithecoid radiation and the collapse of hominoid diversity following a successful radiation during the early and middle Miocene.

The importance of clade interactions in explaining variation in speciation and extinction rates through time has been demonstrated in other groups using paleontological data (Condamine et al., 2019; Pires et al., 2017; Silvestro et al., 2015). The approaches used in those studies can be applied to the catarrhine fossil record to test hypotheses about the nature of the interaction between hominoids and cercopithecoids. For example: Did cercopithecoids actively displace hominoids starting in the late Miocene or passively replace them? Do the differences between hominids and hylobatids in diversification dynamics detected here indicate variation in how hominoids and cercopithecoids interacted? Integration of paleontological and neontological estimates of macroevolutionary rates, which provide different perspectives on diversification dynamics (Silvestro et al., 2018), will also be important. The rich catarrhine fossil record offers an important case study for linking recent evolutionary history to variation in macroevolutionary rates among extant species. The factors that have influenced catarrhine diversification over the last 10 million years are likely to have been important in shaping rate variation in platyrrhines and other primate clades, but in different ways.

### 4.2 The macroevolutionary consequences of primate activity patterns

This study aligns with previous investigations in showing that diurnal and nocturnal primate lineages differ in their diversification dynamics (Magnuson‐Ford & Otto, 2012; Santini et al., 2015; Scott, 2018, 2019). This relationship has also been detected across mammals (Upham et al., 2020) and tetrapods (Anderson & Wiens, 2017). Of the rates examined here, only speciation distinguished diurnal and nocturnal species, with diurnal lineages speciating at significantly higher rates. Other studies have found higher net diversification rates in diurnal lineages as well (Santini et al., 2015; Scott, 2018, 2019), but that rate difference was not recovered in the present analysis.

Santini et al. (2015) conducted the only investigation that examined cathemeral species apart from diurnal and nocturnal lineages. Cathemerality is found at low frequency in primates, occurring only in lemurids (*Lemur*, *Hapalemur*, *Prolemur*, and *Eulemur*) and some populations of *Aotus* (Donati & Borgognini‐Tarli, 2006; Santini et al., 2015; Tattersall, 2006). Santini et al. (2015) concluded that cathemeral lineages have much higher rates of speciation and extinction than diurnal and nocturnal lineages, but with very low net rates of diversification (*r* < 0.01). This finding suggests that cathemerality may be uncommon among primates because it increases macroevolutionary volatility. Given high and nearly equal rates of speciation and extinction, small clades of cathemeral species are likely to go extinct early in their histories, before they expand to sizes that are large enough to ensure long‐term persistence (Chevin, 2016; Gilinsky, 1994).

The results of the present study do not corroborate this hypothesis: there is no evidence that cathemeral lineages have relatively high rates of speciation and extinction—and thus high rates of turnover—compared to other primates, or net rates of diversification that are close to *r* = 0 (Figure 3). Instead, cathemeral species have rate estimates that are mostly similar to those of other lineages in their respective clades. As noted above, species of *Eulemur* have slightly elevated speciation and net diversification rates relative to other lemuriforms, but the phylogenetic ANOVAs did not detect any differences between cathemeral and nocturnal lineages. Differences between cathemeral and diurnal lineages were, with one exception, also not significant. The exception occurred in the analysis conducted using the hard‐bounded tree and the Groves sampling fraction: in this case, diurnal lineages had higher speciation rates than cathemeral lineages. This result should not be considered robust, however, given that it was not recovered in the other analyses. The exact relationship between cathemerality and primate diversification remains unclear.

Also unclear is the mechanistic basis for the correlation between activity pattern and macroevolutionary dynamics. Santini et al. (2015) suggested three hypotheses, two of which specify a mediating influence of other traits—sociality, frugivory, seed dispersal, or geographic range—that have direct effects on diversification. These hypotheses have not received support in subsequent analyses (Scott, 2018). The third hypothesis proposes that nocturnal primate diversity is limited by competition with other mammalian clades, whereas the shift to diurnality may have presented anthropoids with greater opportunity to diversify because few other clades of diurnal mammals have established themselves in the arboreal environment (Santini et al., 2015). This hypothesis is difficult to test, but macroecological approaches may reveal differences among primates in the intensity of competition with other vertebrates (e.g., Beaudrot et al., 2013a; Beaudrot et al., 2013b; Kamilar & Beaudrot, 2013).

Another possibility is that diurnal species may be more susceptible to extinction than nocturnal lineages during periods of climatic cooling (e.g., Wu et al., 2017), resulting in greater macroevolutionary volatility over the last 10 million years as diurnal lineages declined and then recovered in response to climatic oscillations (Scott, 2018). Such boom‐bust dynamics should be characterized by high rates of speciation, extinction, and turnover (Caetano et al., 2018; Vasconcelos et al., 2022b), but the present study only detected higher speciation rates in diurnal lineages. It is possible that the factors driving variation among anthropoid clades have obscured the signal for extinction and turnover, with the very low extinction rates of cercopithecoids being a particularly salient counterexample to the hypothesis. Identifying those factors will be important for understanding the link between activity pattern and macroevolutionary dynamics.

Finally, the high speciation rates found in anthropoids may be a by‐product of traits that evolved as anthropoids adapted to diurnality. An obvious candidate is the highly specialized visual system of anthropoids, including enhanced visual acuity and trichromatic vision (Dominy et al., 2003; Heesy & Ross, 2001; Kay & Kirk, 2000; Ross, 2000; Ross & Kirk, 2007). These adaptations have probably resulted in anthropoids being more reliant than other primates on visual cues during social interactions (Allen et al., 2014; Santana et al., 2012; Santana et al., 2013). Arbour and Santana (2017) hypothesized that the diversification of cercopithecoids may have been facilitated by the evolution of complex patterns of facial coloration in many members of the clade (Allen et al., 2014; Santana et al., 2013). Platyrrhines also exhibit substantial diversity and complexity in facial coloration (Santana et al., 2012), and anthropoids are, in general, more variable in head, body, and limb coloration than strepsirrhines (Bell et al., 2021). It is likely that the elaboration of color patterns has served to reinforce boundaries between closely related species following the initiation of allopatric speciation and subsequent secondary contact (Allen et al., 2014), but such elaboration also has the potential to drive speciation, as conspecific populations in different parts of a species' range diverge in response to environmental gradients and interactions with different sets of species. Establishing whether coloration patterns are a consequence or a cause (or both) of high speciation rates will be challenging. Another major question is whether the visual cues involved in mate recognition in anthropoids are more evolutionarily labile than the olfactory or acoustic signals that are presumably more important for nocturnal lineages.

### 4.3 Do cercopithecoids have unusually low rates of extinction?

The high net diversification rates found in cercopithecoids are primarily a function of their very low extinction rates in comparison to other primates. This finding is not new to this study (Arbour & Santana, 2017; Purvis et al., 1995). The mean extinction rate for cercopithecoids estimated from the tree with soft‐bounded constraints and the sampling fraction derived from the taxonomy of Groves (2005) is 0.015 events per lineage per million years. This value implies a mean species duration of 67 million years and a median duration of 46 million years (Marshall, 2017; Raup, 1985). Both of these estimates are biologically implausible. However, recall that tip rates provide a description of a clade's current macroevolutionary dynamics, influenced by events of the recent past (Title & Rabosky, 2019; Vasconcelos et al., 2022a). It is possible that recent cercopithecoid evolutionary history has been characterized by low rates of extinction as the modern genera have radiated in response to changes in regional environments and primate extinctions associated with Plio‐Pleistocene climatic fluctuations (Elton, 2007; Elton & Dunn, 2020; Roos et al., 2019), with rapid diversification obscuring the signal of extinction. Nevertheless, underestimation of cercopithecoid extinction rates, here and in other studies, is a concern (Rabosky, 2010, 2016).

**FIGURE 4:**
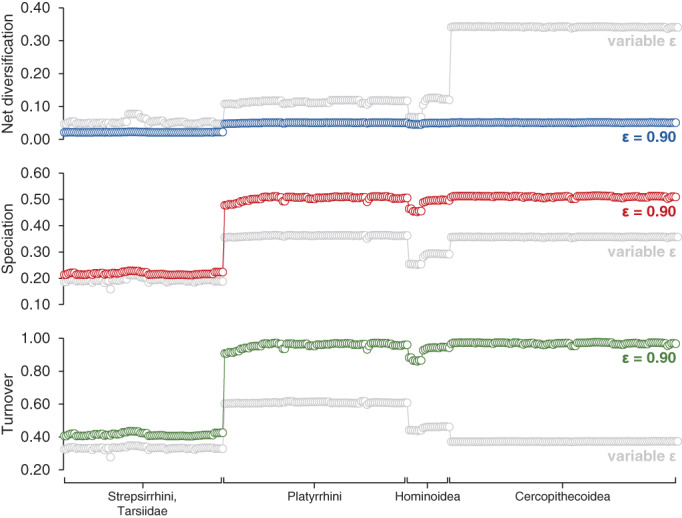
Comparison between tip rates estimated with extinction fraction set to *ε* = 0.9 (color) and the tip rates from the main analysis (gray, variable *ε*); extinction rates are not illustrated because they are perfectly correlated with speciation rate (*μ* = *λε*)

I explored the effects of imposing higher extinction rates on cercopithecoids and other primates by conducting a MiSSE analysis with extinction fractions set to *ε* = 0.9 for all hidden states. This extinction fraction is higher than those estimated in the main MiSSE analysis for other primates (0.60–0.75; Figure 1c), but it is more in line with paleontological estimates (e.g., Gilinsky, 1994; Herrera, 2017a; Marshall, 2017). Fixing the extinction fractions at *ε* = 0.9 has three main consequences: (i) it flattens the rate variation among anthropoids so that cercopithecoids no longer have high net diversification rates relative to other anthropoids; (ii) it magnifies the differences between anthropoids and other primates in rates of speciation and turnover; and (iii) it compresses the differences between anthropoids and other primates in net rates of diversification (Figure 4). Extinction rates are not shown in Figure 4, but they show the same pattern as speciation rates because the two parameters are perfectly correlated (*μ* = *λε*). Thus, the contrast between anthropoids and other primates is robust to increases in relative extinction rates, but cercopithecoids lose their distinctively high net diversification rates, and anthropoids are much less variable in general.

Ultimately, evaluating the plausibility of the low extinction rates estimated for cercopithecoids will require fossil data. It is worth noting here that we should expect to find higher extinction rates in the cercopithecoid fossil record. Speciation and extinction rates estimated from primate fossil data diverge considerably from those reconstructed from molecular phylogenies, with fossil estimates being higher than phylogenetic estimates (Herrera, 2017a). This disparity is not unique to primates (Marshall, 2017; Silvestro et al., 2018). Silvestro et al. (2018) demonstrated mathematically that the difference is inevitable because different types of speciation and extinction are recognized in paleontology: (i) budding speciation, where an ancestor persists alongside a descendent before one of them goes extinct (true extinction); (ii) anagenetic speciation and extinction, where a single, unbranched lineage is divided into chronospecies based on temporal trends in morphology (pseudospeciation and pseudoextinction), and (iii) bifurcating cladogenesis, where morphological differences between two daughter lineages (true speciation) and their direct ancestor are deemed large enough to recognize the pseudoextinction of the ancestor at the cladogenetic event.

The incompleteness of the fossil record and concomitant phylogenetic uncertainty ensures the presence of pseudospeciation and pseudoextinction to some degree in most paleontological taxonomies. These processes will therefore affect paleontological rate estimates (Silvestro et al., 2018; see also Beaulieu & O'Meara, 2022). In contrast, pseudospeciation and pseudoextinction do not influence the shape of molecular phylogenies and so do not factor into phylogenetic estimates of speciation and extinction rates (Silvestro et al., 2018). A key insight from Silvestro et al.’s analysis is that fossil and phylogenetic rate estimates can differ but still be compatible; both types of data can tell us about different aspects of the same underlying diversity‐generating process. Thus, comparison between compatible sets allows for inferences about the pervasiveness of pseudospeciation and pseudoextinction in a clade's fossil record. This framework can be used to reconcile apparently divergent signals provided by neontological and paleontological data, or, when fossil and phylogenetic estimates are incompatible, help identify factors that may be confounding estimates in one of the data sets (Silvestro et al., 2018). With respect to primates, formally integrating the two approaches should improve macroevolutionary inferences and help resolve the conflicting signals detected in phylogenetic and paleontological data (Herrera, 2017a).

## 5 CONCLUSIONS

This study examined variation in macroevolutionary dynamics across extant primates. Five phylogenetic patterns are evident. First, cercopithecoids have very high net rates of diversification in comparison to all other primates, achieved by combining high speciation rates with very low extinction rates. Second, lemuriforms, lorisiforms, and tarsiids are indistinguishable from each other and have low rates of speciation, extinction, net diversification, and turnover. Third, platyrrhines exhibit high speciation rates, high extinction rates, and high turnover rates, and they have higher net diversification rates than strepsirrhines and tarsiids. Fourth, hominoids have rates that are mostly intermediate between those of platyrrhines and the strepsirrhine‐tarsiid group. Finally, within hominoids, hylobatids have higher speciation rates and lower extinction rates than hominids, and hylobatids resemble platyrrhines in net diversification rates, whereas hominids are similar to strepsirrhines and tarsiids.

The analysis of tip rates by activity pattern indicates that diurnal lineages speciate at higher rates than nocturnal lineages, but no other rate differences were detected. Cathemeral species could not be distinguished from the other two groups, and thus the relationship between this activity pattern and diversification remains unclear. While activity pattern has some power to explain rate differences among primates, most of the variation is found among diurnal anthropoids, indicating that other sources of rate heterogeneity—for example, clade‐specific traits, biogeographic effects, competition with other clades, and historical contingency—have had a more influential role in shaping recent primate macroevolutionary dynamics.

## AUTHOR CONTRIBUTIONS

**Jeremiah E. Scott:** Conceptualization (lead); formal analysis (lead); investigation (lead); visualization (lead); writing – original draft (lead); writing – review and editing (lead).

## CONFLICT OF INTEREST

The author declares no conflict of interest.

## Supporting information


**Tables S1, S2,** and **S3** Supporting information

## Data Availability

The tip rates estimated for this study are available in the Supporting Information. The phylogenetic trees used in the analysis are from Springer et al. (2012). Activity‐pattern classifications are from Santini et al. (2015).
